# Key Competencies for Adolescent Well-Being: An Intervention Program in Secondary Education

**DOI:** 10.3390/ejihpe15110219

**Published:** 2025-10-25

**Authors:** Pablo Molina Moreno, María del Mar Simón Márquez, María del Carmen Pérez-Fuentes, María del Mar Molero Jurado

**Affiliations:** Department of Psychology, University of Almería, 04120 Almeria, Spain; pmm143@ual.es (P.M.M.); mpf421@ual.es (M.d.C.P.-F.); mmj130@ual.es (M.d.M.M.J.)

**Keywords:** personal competencies, adolescence, intervention program, pre-post intervention

## Abstract

This study examines the effects of an intervention program aimed at enhancing personal competencies in secondary education students, focusing on resilience, emotional intelligence, self-esteem and assertiveness. A descriptive, quasi-experimental design with pre- and post-intervention assessments was employed. A total of 36 first-year secondary education students participated and completed the Resilience Scale, Wong-Law Emotional Intelligence Scale, Rosenberg Self-Esteem Scale and Rathus Assertiveness Scale. Positive associations were observed among resilience, emotional intelligence, self-esteem, and assertiveness at both time points, with the exception of a post-intervention negative correlation between self-esteem and the appraisal of others’ emotions. While emotion use increased significantly following the intervention, no significant changes were observed in the other variables, indicating a limited impact on these specific aspects of mental and emotional health. These findings highlight the relevance of training and promoting personal competencies in secondary school students, since they serve as protective factors against social exclusion, mood disorders (e.g., anxiety and depression), and behavioral problems. Although the program improved the use of emotions, its lack of significant effects in other domains highlights the need for more programs to support adolescents’ holistic development in the academic context.

## 1. Introduction

Adolescence is a critical stage of development, marked by significant biological changes and profound psychological, emotional, behavioral and social transformations. These processes are best understood within established theoretical frameworks that capture the complexity of adolescent development, such as social-emotional learning theory (Abrahams et al., 2019; Schoon, 2021), self-determination theory (Ryan & Deci, 2017, 2024), and Bronfenbrenner’s ecological systems theory (Bronfenbrenner, 1974, 1979).

Moreover, these changes—recognized by the World Health Organization (WHO, 2020) and documented in recent research—present unique challenges for young people in the 21st century (Moyano et al., 2021). At this stage, vulnerability to risk behaviors such as substance use and early onset of sexual activity increases, a highlighted concern in studies by Booker and Dunsmore (2016), M. M. Molero et al. (2022), and Ruan et al. (2019). In addition, social crises or exceptional circumstances, such as the COVID-19 pandemic, have heightened stress, anxiety, and depressive symptoms among adolescents, further undermining their mental health and their capacity to adapt to a rapidly changing environment (Kirbiš, 2023).

Faced with these challenges, the development of personal competencies becomes essential for adolescents, serving not only to foster optimal adaptation but also to preserve their psychological well-being, as suggested by Schoeps et al. (2021), and Sasagawa and Essau (2022). In today’s secondary education context, where adolescents face unprecedented pressures and challenges arising from rapid social and technological evolution, strengthening personal competencies such as resilience becomes essential. This need of promoting healthy psychological adaptation in adolescents, emphasized by Ruvalcaba et al. (2019) and Garaigordobil (2023), underscores the relevance of implementing intervention programs aimed at providing young people with the necessary skills to successfully navigate their complex environment.

Resilience, defined by Stevenson and Zimmerman (2005) as the ability to overcome the adverse effects of exposure to risk and to cope successfully with traumatic experiences, is fundamental to adolescent development. It not only entails avoiding negative trajectories associated with risk but is also recognized as a positive predictor of quality of life in adolescents. Evidence from Simón-Saiz et al. (2018) and Maheri et al. (2019) shows that resilience underpins positive development during childhood and adolescence, playing a crucial role in promoting mental health and well-being, as supported by Liu et al. (2012), and Hu et al. (2015). Additionally, studies such as Anderson et al. (2020), and Gedda-Muñoz et al. (2023), suggest that resilience acts as a protective factor, reducing the likelihood of developing mental health disorders such as depression and anxiety.

Sanders et al. (2015) highlight that high levels of resilience are correlated with better well-being outcomes, including prosocial behaviors, positive peer relationships, and stronger interpersonal skills. Resilience has also been linked to lower levels of internalizing and externalizing symptoms, as documented by Hjemdal et al. (2011), and Skrove et al. (2013). Given that adolescence is a period of heightened receptivity to interventions, programs focused on promoting resilience may be particularly effective. Such programs, as examined by Fenwick-Smith et al. (2018), and Pinto et al. (2021), emphasize the development of key skills including effective coping strategies, mindfulness, recognition, emotional regulation and management, fostering empathic relationships, self-awareness, and personal efficacy. Evidence from these interventions indicates significant improvements in anxiety and depression symptoms, as well as enhanced academic performance (M. M. Molero et al., 2023). Moreover, the interplay between resilience and emotional intelligence (EI), as reported by Adibsereshki et al. (2019), provides adolescents with essential resources to face challenging situations.

EI is defined as the ability to accurately identify and discriminate one’s own emotions and those of others, to express emotional states appropriately, and to regulate them adaptively in order to respond effectively (Gardner, 1995; J. D. Mayer et al., 2008). This ability, fundamental to human development, translates into a more positive outlook on life, greater self-knowledge, contextually appropriate decision making, and better personal and health development, thereby leading to enhanced health and overall psychological well-being (J. Mayer & Salovey, 1997; J. D. Mayer et al., 2008; Mehta & Mehta, 2015). In educational settings, EI is particularly relevant: adolescents with high levels of EI tend to experience greater well-being and satisfaction, as well as increased academic engagement, achievement, and performance (Fuentes et al., 2023; Toscano-Hermoso et al., 2020). EI not only predicts and favors prosocial behavior, but also promotes greater enjoyment in school, elevated academic aspirations, confidence, and self-esteem, while reducing irritability, sadness, and the occurrence of psychological disorders, conduct problems, and school burnout (Benita et al., 2017; Espino-Díaz et al., 2021; Lerkkanen et al., 2018; Miething et al., 2017; M. M. Molero et al., 2021). Chiș and Rusu (2019) further demonstrate that classroom-based EI training significantly improves students’ quality of life and well-being, while reducing engagement in risky behaviors. This evidence underscores the value of integrating targeted methodologies for EI development into school programs.

In the academic context, emotional intelligence (EI) is closely linked to adolescents’ self-esteem, an important association highlighted by Barragán et al. (2021), and further reinforced by Babić and Tomašić (2023), and López-Noguero and Gallardo-López (2023). Adolescence is a critical period for the development of self-esteem, during which young people begin to consolidate feelings of self-love, respect, and confidence (Díaz et al., 2018; Li et al., 2023). Research by Reguera-López-de-la-Osa et al. (2023) highlight the effectiveness of targeted programs to improve EI and self-esteem in adolescents. Self-esteem is essential for mental health at this life stage (Arsandaux et al., 2021); Katsantonis et al., 2023); whereas low levels are associated with reduced and increased risk of social exclusion, bullying, and mood disorders, including anxiety (Arslan, 2019; Li et al., 2023). Rodríguez-Hidalgo and Pantaleón (2019) also explore the role of EI in influencing behaviors such as bullying in adolescents. Programs that foster self-esteem in adolescents, such as those studied by Moulier et al. (2019) and Golshiri et al. (2023), seek to promote well-being, interpersonal relationships, and protective behaviors by focusing on developing skills such as problem solving, critical thinking, empathy, and interpersonal skills. In addition, high levels of self-esteem are also correlated with greater resilience and assertiveness in the classroom, allowing adolescents to face interpersonal difficulties with greater confidence and efficacy (İlhan et al., 2016; M. D. M. Molero et al., 2019). Assertiveness, a key social skill for interpersonal communication highlighted by Koparan et al. (2009), involves standing up for one’s rights and expressing thoughts and emotions clearly and respectfully. This quality not only influences individual confidence and social adaptation, but also psychological well-being (Pfafman, 2017). Assertiveness training programs, such as those investigated by Kriščiūnaitė and Kern (2014), can transform self-image, enhance the fluent expression of ideas, and strengthen self-esteem, interpersonal relationships, and communication skills. Such training is vital for adolescents, as it cultivates personal confidence, improves social communication skills, and teaches individuals to exercise rights with respect towards others, thereby increasing life satisfaction and happiness (Avşar & Alkaya, 2017). Assertiveness skills training, increasingly common today, is especially beneficial for adolescents facing challenges in their interpersonal relationships (Eslami et al., 2016).

A growing body of international research underscores the importance of implementing programs that foster socio-emotional skills during adolescence. For example, Cerit and Şimşek (2021) reported significant improvements in resilience and EI among Turkish adolescents following an intervention program. Similarly, Golshiri et al. (2023) found that training in problem-solving and assertiveness enhanced self-esteem in Iranian adolescents. In the United States, Young et al. (2012) demonstrated that developing communication skills strengthens social and family relationships and increases emotional engagement with school.

While these findings support the effectiveness of interventions targeting specific competencies, no program to date has been identified that integrates all of these dimensions. The present study addresses this gap by implementing a single intervention program that simultaneously develops EI, resilience, self-esteem, assertiveness, and communication skills in secondary school students.

Given the benefits of this type of competencies in the holistic development of adolescents, the main objective of this exploratory pilot study is to examine the effects of an intervention program on personal competencies in Secondary Education students, with a focus on resilience, EI, self-esteem and assertiveness. This objective is explored through the following interrelated hypotheses.

**H1:**  *A positive association between resilience levels and the other competencies assessed (EI, self-esteem, and assertiveness) will be found, in both the pre- and post-intervention measurements.*

**H2:**  *EI scores (particularly in the use of emotions), self-esteem, and assertiveness will significantly increase following the intervention compared to baseline.*

**H3:**  *Self-esteem and assertiveness will show a positive correlation in both the pretest and posttest.*

**H4:**  *Students will show significant improvements in at least one of the competencies assessed (resilience, EI, self-esteem, or assertiveness) after the intervention.*

Taken together, these hypotheses allow for an in-depth and comprehensive framework for examining how the intervention may promote growth and development across multiple dimensions of secondary school students’ personal attributes and competencies.

## 2. Materials and Methods

This descriptive study employed a quasi-experimental design with pre- and post-intervention assessments.

To determine whether missing data were Missing Completely at Random (MCAR), Little’s MCAR test (Little, 1988) was applied to responses from both Time 1 and Time 2.

### 2.1. Participants

The study sample comprised 36 first-year secondary school students. The mean age was 12.47 years (SD = 0.77) for the total sample, 12.83 years (SD = 0.93) for males, and 12.29 years (SD = 0.62) for females. Of the total sample, 66.67% were female (*n* = 24).

An a priori power analysis for a pre–post design was conducted using parameters recommended in standard power analysis procedures (Cohen, 1988; Faul et al., 2009). Assuming a two-tailed significance level of α = 0.05, a desired power of 0.80, and a medium expected effect size (0.50), the required sample size was estimated at 34 participants. The final sample (*N* = 36) therefore provided adequate statistical power to detect medium effects.

### 2.2. Instruments

An ad hoc questionnaire was used to collect participants’ sociodemographic data, including age, sex and academic year.

To assess resilience, the 10-item CD-RISC (Notario-Pacheco et al., 2011) was administered. This validated scale measures resilience—understood as the capacity to face and overcome adversity—across various areas of life. It consists of 10 items rated on a five-point Likert-type scale (0 = Never, 5 = Almost always). The scale demonstrated good reliability, with omega coefficients of ω = 0.80 for the pretest and ω = 0.86 for the posttest.

On the other hand, Wong-Law’s Emotional Intelligence Scale (WLEIS; Wong & Law, 2002) was used to assess emotional skills. This instrument consists of 16 items rated on a Likert-type scale (where 1 is “Strongly Disagree” and 7 is “Strongly Agree”). It assesses EI through four subscales: the Self Emotion Appraisal (SEA), Other Emotion Appraisal (OEA), Use of Emotions (UOE) and Regulation of Emotions (ROE), providing a detailed understanding of the individual’s emotional abilities. Scale reliability, as measured by the omega coefficient, was ω = 0.76 at pretest and ω = 0.86 at posttest for the SEA subscale; ω = 0.79 at pretest and ω = 0.76 at posttest for the OEA subscale; ω = 0.80 at pretest and ω = 0.90 at posttest for the UOE subscale; and ω = 0.79 at pretest and ω = 0.78 at posttest for the ROE subscale. These values indicate good reliability for the scale both before and after the intervention, reinforcing the validity of the EI measures used in this study.

To assess self-esteem, the Rosenberg Self-Esteem Scale (Rosenberg, 1965) was administered. This instrument evaluates self-esteem following a unidimensional model developed for adolescents and adults, measuring feelings of self-respect and self-acceptance. It consists of 10 items rated on a four-point scale ranging from “strongly agree” to “strongly disagree”. In this study, it demonstrated acceptable reliability, with omega coefficients of ω = 0.73 at pretest and ω = 0.71 at posttest.

Finally, the Rathus Assertiveness Scale (Rathus, 1973) was used to measure assertiveness as another personal skill of interest. This instrument measures the degree of assertiveness through 30 items rated on a six-point scale ranging from +3 (Very characteristic of me, extremely descriptive) to −3 (Very uncharacteristic of me, extremely non-descriptive). In the present study, it demonstrated good reliability, with omega coefficients of ω = 0.81 at pretest and ω = 0.77 at posttest.

### 2.3. Procedure

Prior to data collection, informed consent was obtained from the parents or legal guardians of all participants. The purpose of this study, the procedures involved, and the participant’s rights were clearly explained. Confidentiality and protection of participants’ personal data were guaranteed in accordance with current data protection regulations. This research received the ethical approval report from the Bioethics Committee of the University of Almería (Ref: UALBIO2022/005).

Once all the necessary permissions had been obtained, prior to the start of the intervention, a booklet of questionnaires was carefully prepared in line with the established objectives. The school was also contacted and the pretest was administered to the participating students. The questionnaires were administered face-to-face in a classroom specifically designated for this purpose and participants were allotted 30–35 min to complete the questionnaires.

The program was embedded within an existing school initiative, the *Forma Joven* Program, which formed part of the secondary school’s curriculum. The school administration approached the research group after learning about our prior work on fostering personal skills during adolescence. They had identified shortcomings in this area among its students and requested our collaboration in designing and implementing an intervention tailored to their needs.

The intervention was conducted over eight weekly sessions between March and May 2023, each lasting 45 min and held during the students’ tutorial hour on Thursday mornings, starting on March 8 with the first session. The sessions targeted key personal skills, including communication, assertiveness, self-esteem, EI, and resilience. The research team—comprising four members—planned and delivered the sessions in collaboration with the classroom tutors, who actively participated alongside students. This direct involvement enabled tutors to learn specific strategies for developing these skills through observation and practice, contributing both to their ongoing professional development in the socio-emotional domain and to improvements in the classroom climate through the sustained application of these techniques.

Implementation followed a detailed plan that specified the activities for each session, carefully adapted to the profile of the target students. Sessions began with an introductory and familiarization phase to present the skill being developed, clarify objectives, and prepare students for the tasks ahead. This was followed by practical activities designed to promote skill acquisition. Throughout the process, active student engagement and participation were fostered to ensure continuous involvement in the sessions.

The first session focused on developing communication abilities, aiming to enhance adolescents’ capacity for both effective expression and social interaction. To achieve this, participants engaged in imitation and facial mimicry activities, such as reproducing a chain of gestures in a circle or guessing professions in teams.

The second session continued to focus on communication, emphasizing the alignment between oral discourse and gestural expression through storytelling activities. In one of them, we used a wheel with different areas of life (work, school, family, romantic relationships, friendship, introspection, and personal identity). The person whose turn it was had to share a personal experience related to the area that came up, using additive, exemplary, or corrective connectors, and present their story to the group.

The third session addressed the development of assertiveness, with the goal of equipping participants with the skills needed to initiate, maintain, and conclude conversations effectively and respectfully. Structured group activities were used to train specific communication techniques, including respectful argumentation, active listening, clear articulation of ideas, and assertive strategies that promote emotional regulation during interaction.

The fourth session focused on assertiveness in stressful situations and on managing criticism. Building on previous communication skills, this session emphasized more complex contexts requiring emotional regulation and respectful expression in the face of potential conflict. Participatory activities encouraged the acceptance of mistakes, openness to constructive feedback, the ability to apologize, and the maintenance of emotional self-control, thereby strengthening practical social skills for handling challenging situations.

The fifth session targeted the enhancement in self-esteem in adolescents, addressing four core components: self-image, self-concept, self-efficacy, and self-reinforcement. Group dynamics fostered personal reflection and expression, while the sharing of experiences and the recognition of personal and peer strengths cultivated empathy, self-evaluation, positive acknowledgment, and emotional connection. These activities created a safe space for emotional expression and the development of interpersonal bonds.

The sixth session introduced exercises designed to build cognitive and emotional resilience. Activities highlighted the difficulty of directly controlling unwanted thoughts and promoted more functional strategies for emotional regulation. Acceptance-based tasks encouraged reflection on the inevitable and adaptive role of discomfort in daily life. Finally, participants engaged in exercises linking personal experiences to the identification of specific coping resources and emotional management strategies.

The seventh session focused on developing EI, with particular attention to identifying and expressing emotions from an empathetic perspective. Progressive activities involved recognizing basic emotions through facial expressions, interpreting situational cues in varied emotional contexts, and exploring hypothetical scenarios relevant to adolescence. These tasks encouraged self-reflection, group discussion, and the generation of adaptive emotional responses.

The eighth and final session explored emotional regulation, with an emphasis on accepting discomfort and managing difficult emotions. Activities invited participants to reflect on the limitations of avoidance strategies and to explore more adaptive approaches grounded in acceptance and coping. Group dynamics enabled experiential learning about the impact of resisting emotions versus allowing them to be present.

At the end of the intervention, a post-test evaluation was administered to assess its effectiveness.

**Figure 1:**
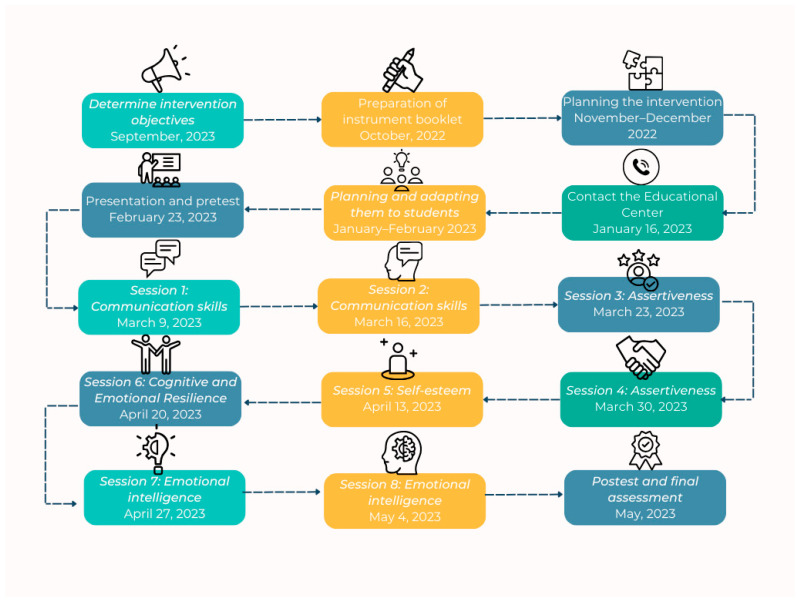
Intervention program flowchart.

The intervention was designed and implemented following a structured sequence, from initial planning to final evaluation. Figure 1 presents a flowchart outlining the process phases, which included pre-intervention tasks, the delivery of eight thematic sessions tailored to the students’ profile, and both pretest and posttest assessments.

### 2.4. Data Analysis

First, the assumption of normality was tested using Kolmogorov–Smirnov (n ≥ 30; Vovk, 1993), which confirmed that the data did not clearly conform to a normal distribution. Therefore, nonparametric tests were employed.

To examine the associations among resilience, EI, self-esteem, and assertiveness, bivariate correlation analyses were conducted using Spearman’s rho coefficient at both measurement points (pre- and post-intervention). In addition, heatmaps were created to provide an intuitive visual comparison of the strength and direction of correlations at both time points, facilitating the identification of consistent associations as well as potential changes in interrelationships between variables after the intervention.

Subsequently, to assess whether there were variations in the scores of the study variables before and after participation in the program, the Wilcoxon test was applied. The effect size was estimated using the rank-biserial correlation measure (rrb) with its corresponding 95% confidence interval, considering the following cut-off points: 0.10 small, 0.30 medium, and 0.50 large (Coolican, 2009). This metric is considered appropriate for nonparametric tests and allows for a clear interpretation of the magnitude of change between conditions (Kerby, 2014).

The reliability of the instruments was examined using McDonald’s omega coefficient (McDonald, 1999), following the recommendations of Ventura-León and Caycho (2017). Data were analyzed using SPSS v24.0 (IBM Corp., 2016). In addition, JASP v0.18.1 (JASP Team, 2023) was used as a complementary tool for estimating the rank-biserial correlation.

## 3. Results

First, the pre- and post-intervention correlations among resilience, EI (dimensions: evaluation of one’s own emotions, evaluation of others’ emotions, use of emotions, and emotion regulation), self-esteem and assertiveness are presented (Table 1).

In both measurements (pre- and post-intervention), resilience showed positive associations with appraisal of own emotions, use of emotions, emotion regulation, self-esteem and assertiveness.

**Figure 2:**
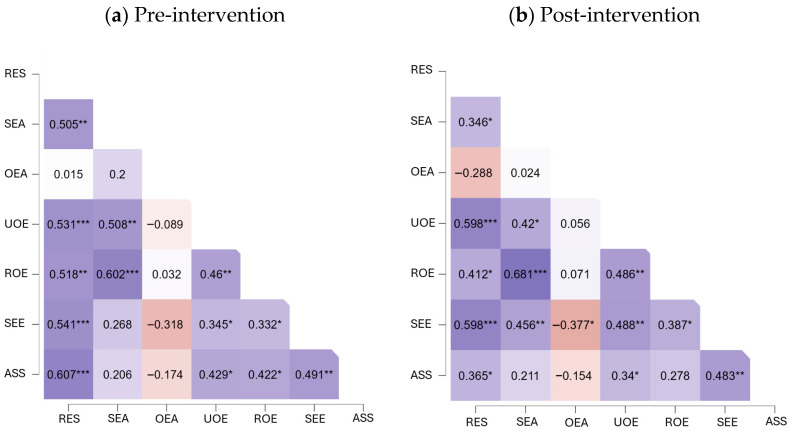
Heatmaps of the correlation matrices for study variables at pre-intervention (**a**) and post-intervention (**b**) time points. Notes: RES, Resilience; SEA, Evaluation of own emotions; OEA, Evaluation of others’ emotions; UOE, Use of emotions; ROE, Regulation of emotions; SEE, Self-esteem; ASS, Assertiveness. Colors represent the magnitude and direction of the correlations (Spearman’s rho), with darker tones indicating stronger associations. *** *p* < 0.001, ** *p* < 0.01, * *p* < 0.05.

As visual support for the correlational results, Figure 2 shows the correlation matrices corresponding to the pre-intervention (Figure 2a) and post-intervention (Figure 2b) moments. This representation allows for a clearer observation of certain patterns of change or stability in the relationships between variables that complement the analyses presented in Table 1.

On the other hand, in the pre-intervention measurement, self-esteem was positively associated with resilience, use of emotions, emotion regulation, and assertiveness. In the post-intervention measurement, self-esteem remained positively associated with resilience, evaluation of one’s own emotions, use of emotions, and emotion regulation; however, a negative correlation was observed with the evaluation of others’ emotions (Table 2).

**Figure 3:**
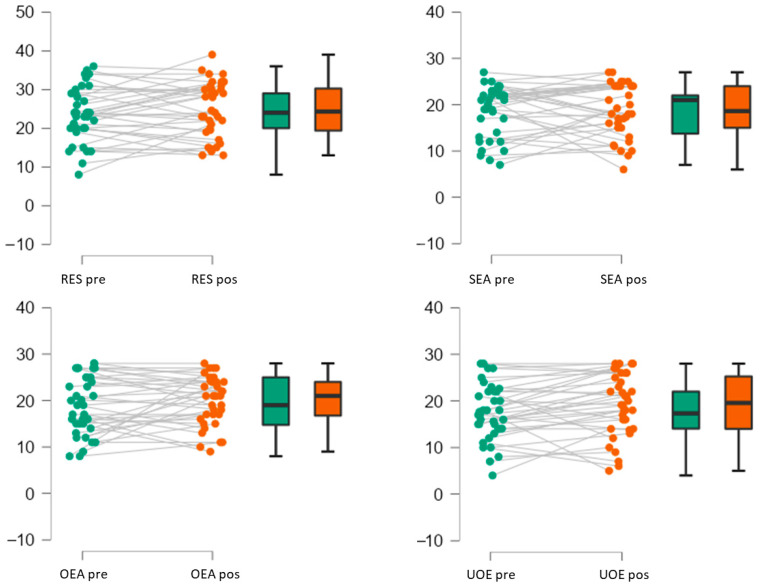

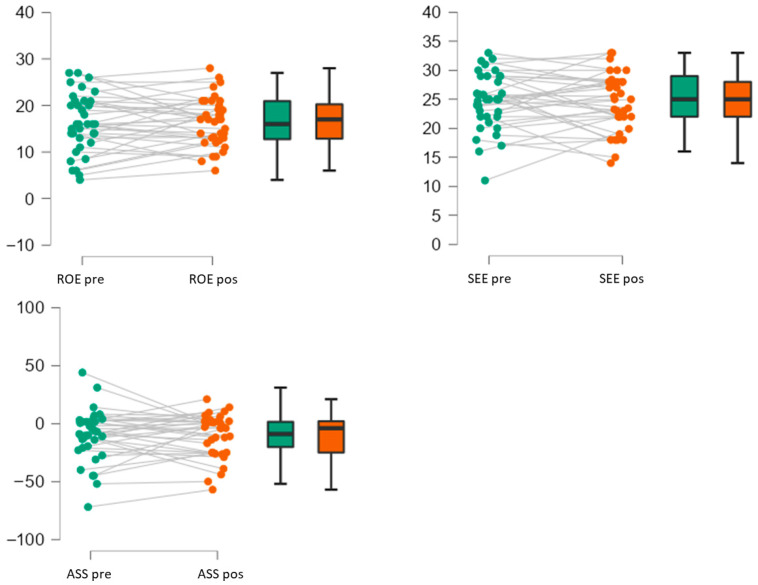
Raincloud Plots ^1^. ^1^ Notes: Displays the individual cases (colored dots), box plots, and densities for each measure. Cases from both measures are connected with individual lines. The x-axis and colors represent the measures (pre-intervention measure in green and post-intervention measure in orange), and the y-axis represents the dependent variable. In the box plots, the bold black line indicates the sample median, the hinges correspond to the 25th and 75th quantile, and the whiskers extend to 1.5 interquartile ranges beyond the hinges.

The results showed that, after the intervention, there are significant changes in the use of emotions, with mean scores increasing from pre-intervention (M = 17.58, SD = 6.05) to post-intervention (M = 19.13, SD = 6.73) (W = 173.50, *p* = 0.035). However, no significant differences were found in resilience, appraisal of one’s own emotions, appraisal of others’ emotions, emotion regulation, self-esteem, or assertiveness. Spearman correlation coefficients revealed negative correlations between time and these variables, suggesting that the intervention did not produce significant effects on these specific aspects of mental and emotional health (Figure 3).

## 4. Discussion

The purpose of this study was to analyze the effects of a Personal Competencies program in adolescents, focusing on key variables such as resilience, EI, self-esteem and assertiveness. Developing personal competencies during this stage is essential, since adolescents need to strengthen these skills in order to adapt effectively and preserve their psychological well-being.

Regarding our first hypothesis, the results revealed a positive association between resilience, EI variables (evaluation of one’s own emotions, use of emotions and emotion regulation), self-esteem and assertiveness both before and after the intervention. These associations highlight the importance of implementing programs that incorporate key skills—such as mindfulness, emotional recognition, emotional regulation and management, personal acceptance, and social support—to foster empathic and healthy interpersonal relationships (Fenwick-Smith et al., 2018; M. D. M. Molero et al., 2019; Pinto et al., 2021) and to develop effective coping strategies for managing stressful and challenging situations (Adibsereshki et al., 2019).

In relation to the second hypothesis, positive associations were observed between the different EI subscales and the variables of self-esteem and assertiveness at both pre- and post-intervention. This may be explained by the fact that adolescents with higher levels of EI tend to maintain a more positive mood and are able to manage negative emotions in challenging situations by selecting appropriate emotional strategies. Such regulation facilitates the development of a healthy self-concept and self-esteem, reduces stress, and enhances emotional responsiveness (Barragán et al., 2021). Furthermore, assertive skills have been shown to improve social communication, enable individuals to exercise their rights with respect for others, and ultimately increase life satisfaction and happiness (Avşar & Alkaya, 2017).

Regarding the third hypothesis, significant positive associations were found between self-esteem and assertiveness both before and after the intervention, implying that higher levels of self-esteem are associated with higher levels of assertiveness and vice versa. Notably, this association persisted after the intervention, suggesting consistency in these results over time and across conditions. These findings are consistent with those reported by Golshiri et al. (2023) and İlhan et al. (2016). In general, high self-esteem can increase self-confidence, forming the fundamental basis for assertiveness. From an ecological perspective, Bronfenbrenner’s systems theory (Bronfenbrenner, 1974, 1979) provides a framework for understanding how this relationship may be shaped by multiple interrelated contexts. For example, the microsystem—comprising family, school, and peer group—directly influences the development of self-esteem and social skills. Similarly, interactions between these contexts (mesosystem), broader social and cultural norms (macrosystem), and life changes or transitions (chronosystem) can all modulate how adolescents construct their self-concept and express assertiveness. Thus, the observed association between self-esteem and assertiveness can be understood as the product of a dynamic interaction between the individual and their ecological environment.

Finally, the students who participated in the intervention program in personal competencies obtained significant improvements in the use of emotions, with an increase in mean scores in the post-intervention compared to the pre-intervention. This suggests that the program was effective specifically in enhancing the use of emotions, a skill fundamental to the personal and social development of adolescents. Strengthening this ability can yield substantial benefits in various aspects of daily life, including interpersonal relationships, problem-solving, and stress management (Chiș & Rusu, 2019; M. M. Molero et al., 2021). In line with the theory of social-emotional competencies, these skills enable people to express, manage, and understand their thoughts, emotions, and behaviors in everyday situations, as well as interact with others and adapt to changing situations (Abrahams et al., 2019; Schoon, 2021).

The present results have important implications for students’ holistic development and their ability to cope effectively with life challenges. In this regard, Self-Determination Theory (SDT; Ryan & Deci, 2017) is particularly relevant, as it posits that well-being and personal motivation depend on the satisfaction of three basic psychological needs: autonomy, competence, and relatedness. Strengthening emotional competencies can help meet these needs, thereby promoting a more positive self-perception and more adaptive functioning across different life contexts (Ryan & Deci, 2024).

### Limitations and Future Directions

Despite the valuable findings observed, it is important to acknowledge this study’s limitations. The first concerns the limited duration of the intervention; with a greater number of sessions over a longer period, the program could have addressed the development of competencies more comprehensively, thereby enabling more substantial and lasting changes in participants’ behavioral repertoire over the long term.

Another major limitation of this study was the absence of a control group, which clearly prevents establishing direct causal relationships between the intervention and the changes observed. This was due to organizational reasons, as the intervention was institutionally promoted by the guidance team and implemented with students from the same grade. In this context, excluding part of the student body was not feasible without compromising equity in access to a program aimed at emotional well-being and the development of personal skills. Despite this limitation, the pre–post design allowed for the detection of intra-subject variations and provided relevant preliminary data to guide future research with more robust experimental designs that explicitly incorporate a control group.

The small sample size represents another important limitation, as it restricts the generalizability of the findings. Although the sample size may be considered limited, it falls within an acceptable range for pilot or feasibility studies in educational contexts (Beets et al., 2021), particularly when considering the characteristics of the participants, the complexity of the intervention, and the application context. While the total number of participants could have been higher, the program was delivered to large groups of students within the classroom. Although this format facilitated implementation from an organizational standpoint, it may have limited individual engagement for some students in certain activities.

A further limitation was the absence of qualitative data capturing the subjective experiences and contextual factors perceived by participating students. Such information could have enriched the evaluation of the program by providing more in-depth feedback on its effectiveness from the students’ perspective.

Finally, negative correlations were identified between some of the variables assessed before and after the intervention. This may reflect a smaller gain among participants with higher initial scores, underscoring the need to consider differentiated strategies and longitudinal assessments to explore the stability of changes over time.

Another limitation concerns the limited impact of the program on some of the targeted dimensions, such as resilience, evaluation of one’s own and others’ emotions, emotion regulation, self-esteem, and assertiveness. While significant improvements were observed in the use of emotions, no comparable effects emerged in these other areas. This contrasts with previous studies reporting gains in resilience, self-esteem, and assertiveness following similar interventions (Fenwick-Smith et al., 2018; Golshiri et al., 2023; Moulier et al., 2019). Such findings highlight the need for future studies to explore whether a longer duration, alternative methodologies, or adaptations to different cultural and educational contexts may produce more robust effects across a broader set of competencies.

Refining the methodology through a greater number of intervention sessions, increasing the total sample size to achieve greater population representativeness, and implementing exercises in smaller groups could provide a more comprehensive and nuanced understanding of the effects of the proposed intervention. Furthermore, including a control group in future studies would strengthen the ability to validate the program’s impact more robustly, enabling clearer conclusions about causal relationships. Future research should also incorporate qualitative techniques, such as focus groups or open-ended questions, to capture students’ perspectives and provide a deeper understanding of the program’s effects. Long-term follow-up is likewise recommended.

In the present study, we collected and analyzed the results of implementing an intervention program in a sample of ESO students. Moving forward, new objectives and research lines could be proposed, leading to further studies that either incorporate and refine the activities and tools already used or introduce new proposals to increase variability. Such interventions could also be applied to other populations and cultural contexts.

## 5. Conclusions

Adolescence is a crucial stage in the development of young people and requires particular attention to strengthening personal competencies that enable them to face the challenges and changes inherent to this period of learning and growth. This study underscores the importance of training and promoting competencies such as resilience, emotional intelligence (EI), self-esteem, and assertiveness in high school students, as these play a fundamental role in building a healthy future for adolescents. The personal competencies targeted in the intervention program equip adolescents with greater skill in distinguishing between their own emotions and those of others, as well as enhanced capacity for perspective-taking, decision-making, adapting to environmental demands, and improving communication and interpersonal interaction skills. Collectively, these competencies serve as positive predictors of adolescent health, quality of life, and psychological well-being, fostering confidence and a sense of belonging. Moreover, they act as protective factors against social exclusion, mood disorders such as anxiety and depression, and behavioral problems including substance abuse and violent conduct.

This intervention program led to significant improvements in the use of emotions among the participating adolescents; however, no significant effects were found in other dimensions, such as resilience, emotion appraisal or emotion regulation. These results indicate that promoting personal competencies in adolescents, such as those addressed in this study, can yield significant benefits; however, the effectiveness of an intervention program may vary depending on certain factors. Given the absence of long-term follow-up, it is possible that the significant differences observed in the use of emotions represent a temporary effect. This limitation may be related to the lack of reinforcement sessions after the intervention, which could facilitate greater consolidation of the effects achieved.

Given the relevance of this type of intervention for the holistic development of adolescents, designing and implementing more programs that focus on developing these competencies within the academic context is suggested.

## Figures and Tables

**Table 1:** Correlation matrices between study variables (pre- and post-intervention).

	RES Pos	SEA Pos	OEA Pos	UOE Pos	ROE Pos	SEE Pos	ASS Pos	
RES pre	-	0.35 *	−0.29	0.60 ***	0.41 *	0.60 ***	0.36 *	RES pos
SEA pre	0.50 **	-	0.02	0.42 *	0.68 ***	0.46 **	0.21	SEA pos
OEA pre	0.01	0.20	-	0.06	0.07	−0.38 *	−0.15	OEA pos
UOE pre	0.53 ***	0.51 **	−0.09	-	0.49 **	0.49 **	0.34 *	UOE pos
ROE pre	0.52 **	0.60 ***	0.03	0.46 **	-	0.39*	0.28	ROE pos
SEE pre	0.54 ***	0.27	−0.32	0.34 *	0.33 *	-	0.48 **	SEE pos
ASS pre	0.61 ***	0.21	−0.17	0.43 *	0.42 *	0.49 **	-	ASS pos
	RES pre	SEA pre	OEA pre	UOE pre	ROE pre	SEE pre	ASS pre	

Notes: RES, Resilience; SEA, Evaluation of own emotions; OEA, Evaluation of others’ emotions; UOE, Use of emotions; ROE, Regulation of emotions; SEE, Self-esteem; ASS, Assertiveness. Correlations between variables: before intervention (left matrix–bottom), after intervention (right matrix–top). *** *p* < 0.001, ** *p* < 0.01, * *p* < 0.05.

**Table 2:** Paired samples *t*-test (Wilcoxon signed-rank test).

	Pre		Post		W	z	*p*	r_rb_	95% CI for r_rb_	
	M	SD	M	SD					Lower	Upper
Resilience	23.59	7.06	24.77	7.19	234.50	−1.077	0.285	−0.212	−0.537	0.169
SEA	18.61	5.50	18.83	5.82	291.50	−0.652	0.519	−0.125	−0.462	0.244
OEA	18.92	6.25	19.95	5.28	233.00	−0.580	0.568	−0.117	−0.474	0.272
UOE	17.58	6.05	19.13	6.73	173.50	−2.120	0.035	−0.417	−0.680	−0.058
ROE	16.21	6.31	16.43	5.28	301.00	−0.229	0.824	−0.044	−0.401	0.323
Self-esteem	24.59	4.91	24.50	4.93	280.50	0.000	1.000	0.000	−0.372	0.372
Assertiveness	−10.41	22.50	−10.77	18.66	319.00	0.066	0.954	0.013	−0.352	0.374

Note: RES, Resilience; SEA, Evaluation of own emotions; OEA, Evaluation of others’ emotions; UOE, Use of emotions; ROE, Regulation of emotions; SEE, Self-esteem; ASS, Assertiveness; r_rb_, Rank-Biserial correlation.

## Data Availability

The data presented in this study are available on request from the corresponding author.
